# Mycosynthesis of silver nanoparticles using marine fungi and their antimicrobial activity against pathogenic microorganisms

**DOI:** 10.1186/s43141-023-00572-z

**Published:** 2023-11-21

**Authors:** Manar A. Basheer, Khaled Abutaleb, Nermine N. Abed, Amal A. I. Mekawey

**Affiliations:** 1 National Authority for Remote Sensing and Space Sciences (NARSS), 23 Joseph Tito Street, El-Nozha El-Gedida, Cairo, 1564 Egypt; 2 Agricultural Research Council, Natural Resources and Engineering (ARC-NRE), Pretoria, 0001 South Africa; 3 School of Animal, Plant and Environmental Sciences, University of Witwatersrand, Private Bag X3, Johannesburg, 2050 South Africa; 4 Faculty of Science (Girls Branch), Al-Azhar University Egypt, Nasr City, Cairo11884 Egypt; 5 The Regional Center of Mycology and Biotechnology, Al-Azhar University, Cairo, 4434010 Egypt

**Keywords:** Silver nanoparticle, Antimicrobial activity, Marine fungi, Myco-remediation

## Abstract

**Objectives:**

At the present time, there is a persistent need to get rid of environmental contaminants by eco-friendly, sustainable, and economical technologies. Uncontrolled disposal practices of domestic and industrial solid and liquid wastes led to water pollution which has negative impacts on public health, environment, and socio-economic development. Several water-borne diseases are spreading man to man by microorganisms such as pathogenic bacteria. For the protection of water bodies, all wastewater from various sources should be managed and remediated properly. Myco-remediation is a form of bioremediation in which fungi are used to get rid of contaminants. Fungi are attractive agents for the biosynthesis of nanoparticles especially silver nanoparticles (AgNPs) which are considered one of the most widely utilized nanoparticles because of their unique characteristics such as antibacterial, antiviral, antifungal, and anti-inflammatory properties.

**Methods:**

This study uses silver nitrate and supernatants of four marine fungi; *Penicillium simplicissimum*, *Aspergillus terreus*, *Aspergillus japonicus*, and *Aspergillus oryzae* for extracellular biosynthesis of silver nanoparticles and to evaluate its activity against different pathogenic microorganisms. These nanoparticles may subsequently be applied for the treatment or nano-bioremediation of microbial contaminants in water bodies and improve water quality.

**Results:**

Silver nanoparticles were synthesized and the results revealed that spherical and well-dispersed nanoparticles of different sizes were formed with sizes ranging between 3.8 and 23 nm. Characterization results approved the existence of stable nanocrystalline elemental silver. Antibacterial activity results revealed that AgNPs can be used as a powerful antimicrobial agent for several pathogenic bacteria, yeast, and fungi. Among the biosynthesized NPs mediated by the four marine fungi, AgNPs mediated by *A. japonicus* (5 mM) had the highest antibacterial activity, while AgNPs mediated by *Penicillium simplicissmum* (8 mM) had the highest antifungal activity.

**Conclusion:**

Marine fungi can biosynthesize stable AgNPs that exhibit potent antimicrobial activity against a variety of pathogens.

## Background

Environmental pollution is a pressing global concern with far-reaching implications for ecosystems and human health. As industries and urbanization continue to grow, so do the challenges associated with pollutants in our air, soil, and water. Addressing these environmental issues necessitates innovative and sustainable approaches to remediate contaminants effectively.

This paper explores emerging techniques in environmental remediation, focusing on bioremediation, nanotechnology, and myco-remediation. These approaches harness the power of biological and nanomaterial-based processes to offer eco-friendly and cost-effective solutions. In this background, we provide an overview of these techniques and their significance in contemporary environmental research.

Bioremediation is an eco-friendly and cost-effective technique that utilizes microorganisms to reduce or eliminate contaminants in water, soil, or air. It has gained significant public approval due to its ability to produce bioactive end products and its in-situ applicability [3, 28, 26]. Traditional physio-chemical methods, while effective, are not easily applicable on a large scale, unlike bioremediation, which has been documented as an environmentally friendly and economical approach for treating water pollution [8, 21, 30, 31].

Nanotechnology involves the use of nanoparticles (NPs), synthesized particles less than 100 nm in size, as efficient, cost-effective, and eco-friendly alternatives for environmental remediation. The biosynthesis of nanoparticles has seen significant growth, leading to the production of novel, eco-friendly, and cost-effective materials with diverse applications [15, 22, 35] study confirms the superiority of nanotechnology over other methods for addressing environmental contaminants. Chemical synthesis of nanoparticles, using hazardous and combustible compounds, poses environmental concerns. In contrast, biosynthesis offers a secure, environmentally friendly, and sustainable alternative, with a one-step production process facilitated by biological extracts containing reducing and stabilizing chemicals [12].

Myco-remediation involves using fungi to remediate toxic wastes by leveraging extracellular enzymes to facilitate natural ecological succession [1, 19]. Fungi, with their high metal tolerance and abundant extracellular proteins, are attractive agents for biological nanoparticle synthesis, providing stability to the nanoparticles [16]. Controlling fungal metabolism parameters, such as time, temperature, pH, and biomass quantity, allows to production of nanoparticles with the desired size and morphology.

The integration of nanomaterials and bioremediation has significant potential to create a more effective, efficient, and sustainable remediation process [6]. Various fungal strains, including *Fusarium*, *Aspergillus*, *Verticillium*, and *Penicillium*, have been used for metal nanoparticle synthesis (NPs) [25, 2].

Silver nanoparticles (AgNPs) are considered among the most attractive nanomaterials, with broad applications in biomedicine. Their antimicrobial properties make them valuable for various manufacturing processes. Notably, AgNPs are non-toxic to humans at extremely low concentrations [11]. AgNPs have diverse mechanisms of action against pathogenic bacteria, such as *Escherichia coli* and *Staphylococcus aureus*, allowing them to penetrate cell walls and cell membranes and affect intracellular components. This antimicrobial activity holds promise for medical applications in treating infectious diseases [5].

The aim of this research is to assess the effectiveness of biosynthesized AgNPs produced by four marine fungi isolated from Qarun Lake, Fayoum, Egypt, namely *Penicillium simplicissimum*, *Aspergillus terreus*, *Aspergillus oryzae*, and *Aspergillus japonicus*. These AgNPs will be evaluated for their antimicrobial activity against a spectrum of pathogenic microorganisms, including *Escherichia coli, Klebsiella pneumoniae, Proteus vulgaris, Salmonella typhi* as a Gram-negative bacteria, *Enterococcus faecalis, Methicillin Resistance Staphylococcus aureus* (MRSA), *Staphylococcus hominis,* and *Staphylococcus epidermidis* as a Gram-positive bacteria, two species of pathogenic yeast (*Candida albicans, Candida tropicalis*), and three species of pathogenic fungi (*Aspergillus niger, Penicillium expansum, Rhizopus oryzae*). This study seeks to investigate the potential of these fungal communities and their biologically synthesized nanoparticles for nano-bioremediation of microbial contaminants, ultimately contributing to the enhancement of water quality. The findings presented here will advance our understanding of the environmental applications of nanomaterials and bioremediation techniques, providing insights into their effectiveness and sustainability.

## Methods

### Isolation and identification of marine fungi

Four distinct species of marine fungi were successfully isolated from water samples obtained from Lake Qarun in Fayoum, Egypt. The water samples were meticulously preserved in sterile bottles at a temperature of 4 °C. Subsequently, a volume of 50 mL from each water sample was spread onto Malt Extract Agar plates (containing Malt extract, 20.0 g; Bacteriological Peptone, 1.0 g; glucose, 20 g; Agar, 20.0 g). The antibiotic Chloramphenicol was used as an antibacterial agent. The Agar plates were incubated at 28 °C for 7 days. The inoculated agar plates were examined daily for fungal growth followed by purification. The isolated fungi were identified in the Regional Centre for Mycology and Biotechnology (RCMB), Al-Azhar University, Egypt based on their morphological and reproductive characteristics [4]. For more precise identification, the isolated fungi were identified by 18S-rDNA sequence analysis for molecular identification. A nucleotide sequence database search was conducted using the BLAST program from NCBI GenBank to evaluate the phylogenetic tree of the isolated fungi. The phylogenetic tree was constructed using MEGA 11 software.

### Biomass preparation

Twenty grams of fungal biomass were grown on malt extract broth at 28 °C on a rotary shaker (120 rpm) for 4 days. After that, the biomass was filtered and then washed with distilled water. The biomass was placed in individual flasks containing 100 ml distilled water and incubated for 24 h. The biomass was filtered, and the cell filtrate was collected and used for the biosynthesis of AgNPs following the method outlined by Wang et al. [34].

### Biologically synthesis of silver nanoparticles (NPs)

In the context of extracellular biosynthesis of AgNPs, a volumetric ratio of 1:1 was employed, entailing the mixing of 20 mL of cell filtrate with 20 mL of a 1-mM AgNO_3_ solution. A reaction mixture without AgNO_3_ was used as a control. The prepared solutions were incubated at 28 °C for 24 h in the dark until the color change [18].

### Optimization of reaction parameters for biosynthesis of AgNPs

To determine the best incubation time, 20 ml of 1 mM of AgNO_3_ was mixed with 20 ml of cell filtrate in a 250-ml Erlenmeyer flask and agitated in the dark at a pH of 7 and a temperature of 37 °C. Optical density measurements were conducted at equal time intervals to determine the optimum incubation time and wavelength for AgNPs synthesis. An aqueous solution of AgNO_3_ ranging from 1 to 10 mM was added to the fungal filtrate, then incubated at different temperature conditions (15, 20, 28, 37, and 50 °C) and at pH ranging from 5 to 10 for optimization of substrate concentration, temperature, and pH. The absorption spectrum of the medium containing the silver ions was measured from 200 to 800 nm. A sample of 1 ml was withdrawn at equal time intervals and the optical absorbance was measured at the optimum wavelength.

### Characterization of nanoparticles (NPs)

Characterization was executed by various techniques. Including visual observation, UV–Visible Spectroscopy, energy-dispersive X-ray (EDX) of Scanning Electron Microscopy (SEM), X-ray diffraction (XRD) of TEM, Transmission Electron Microscopy (TEM), zeta potential, and Fourier transform infrared (FT-IR) analysis [9].

### Antimicrobial activity of the biosynthesized AgNPs

The antimicrobial activity of the biosynthesized AgNPs was investigated against 8 species of pathogenic Gram-negative bacteria; *Escherichia coli, Klebsiella pneumoniae, Proteus vulgaris, Salmonella typhi*, and Gram-positive bacteria *Enterococcus faecalis, Methicillin Resistance Staphylococcus aureus* (MRSA), *Staphylococcus hominis,* and *Staphylococcus epidermidis*, two species of pathogenic yeast (*Candida albicans, Candida tropicalis*), and three species of pathogenic fungi (*Aspergillus niger, Penicillium expansum, Rhizopus oryzae*) (obtained from Antimicrobial unit, RCMB, Al-Azhar university, Egypt) using agar well diffusion methods [9]. Wells of 6 mm diameter were cut on the plates using a cork porer and 50 μl of aqueous solution of AgNPs of the most effective concentration (2, 5, and 8 mM) were added to each well. The plates were left in the refrigerator for 30 min and then incubated overnight at 37 °C for bacteria and yeast and 28 °C for fungi for 24 h. Diameters of inhibition zones were meticulously measured after 24–48 h of incubation.

### Statistical analysis

The experimental outcomes were meticulously conducted in triplicate, with subsequent computation of both the means and standard errors. Significance levels were established at *p* < 0.05 through the application of Tukey’s HSD (Honest Significant Difference) post hoc test.

## Results and discussion

### Isolation and identification of marine fungi

**Fig. 1:**
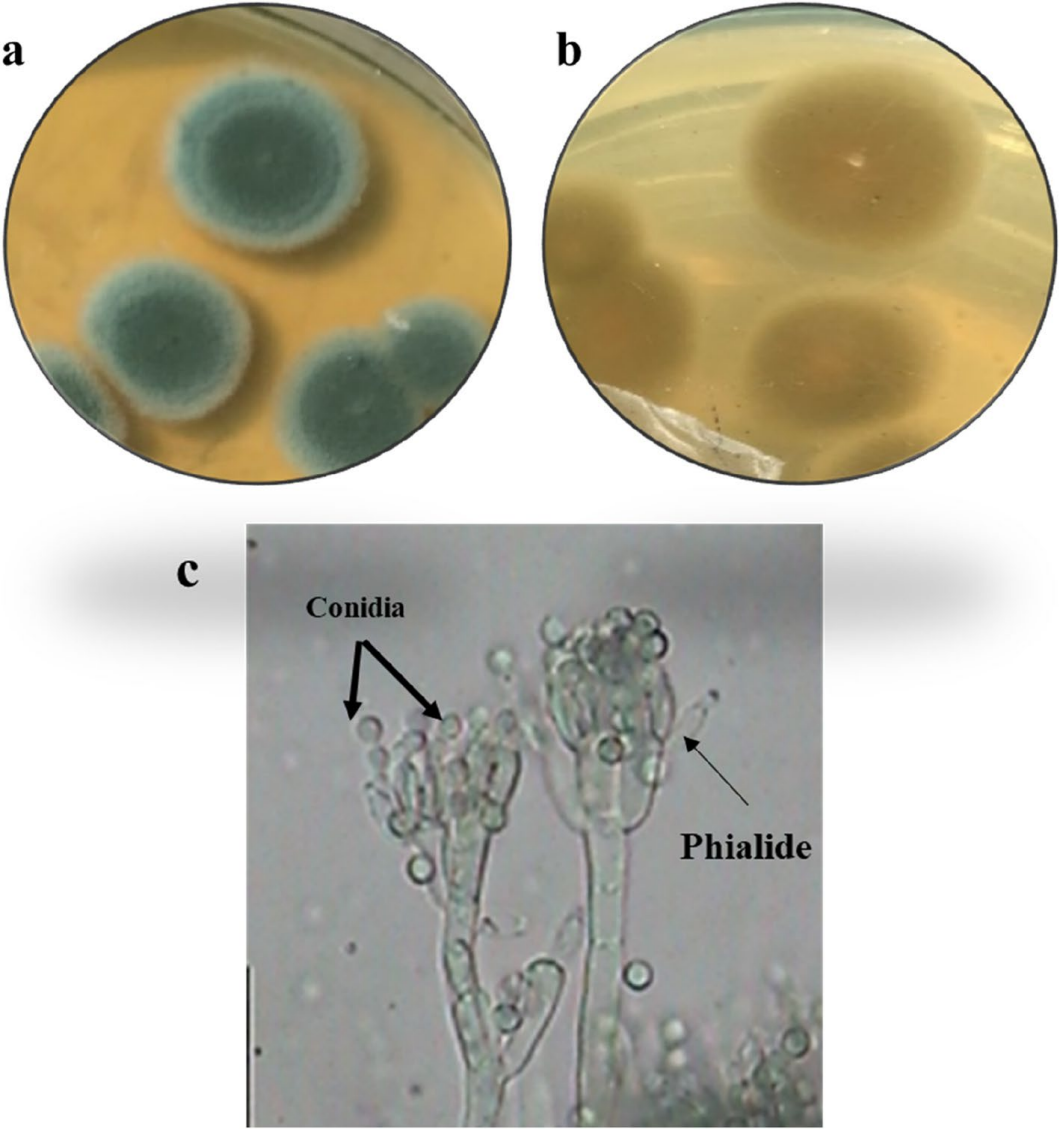
*Penicillium simplicissimum* **a** colony on MEA, **b** colony reverse, **c** microscopic characteristics

**Fig. 2:**
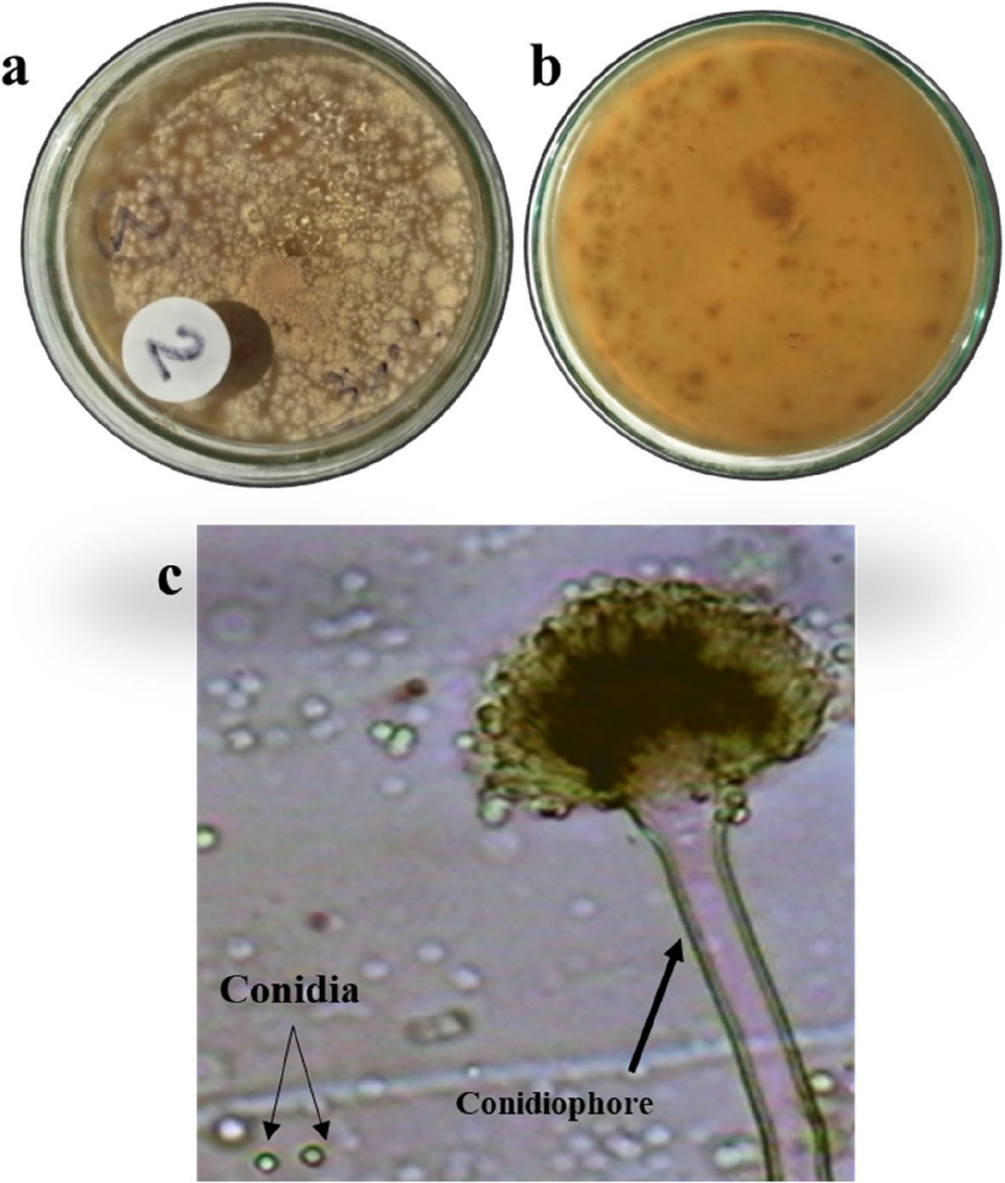
*Aspergillus terreus* **a** colony on MEA, **b** colony reverse, **c** microscopic characteristics

**Fig. 3:**
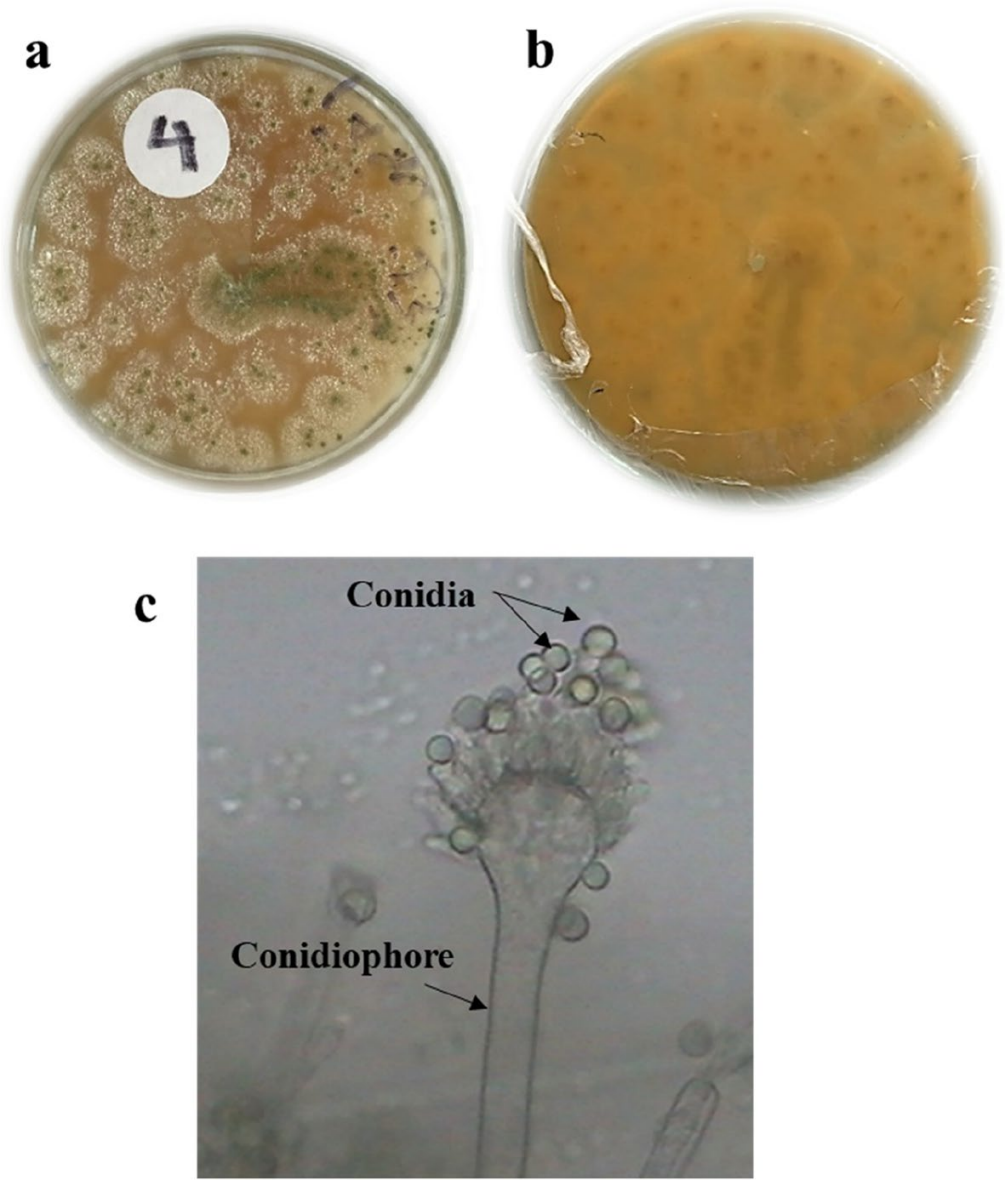
*Aspergillus oryzae* a colony on MEA, **b** colony reverse, **c** microscopic characteristics

**Fig. 4:**
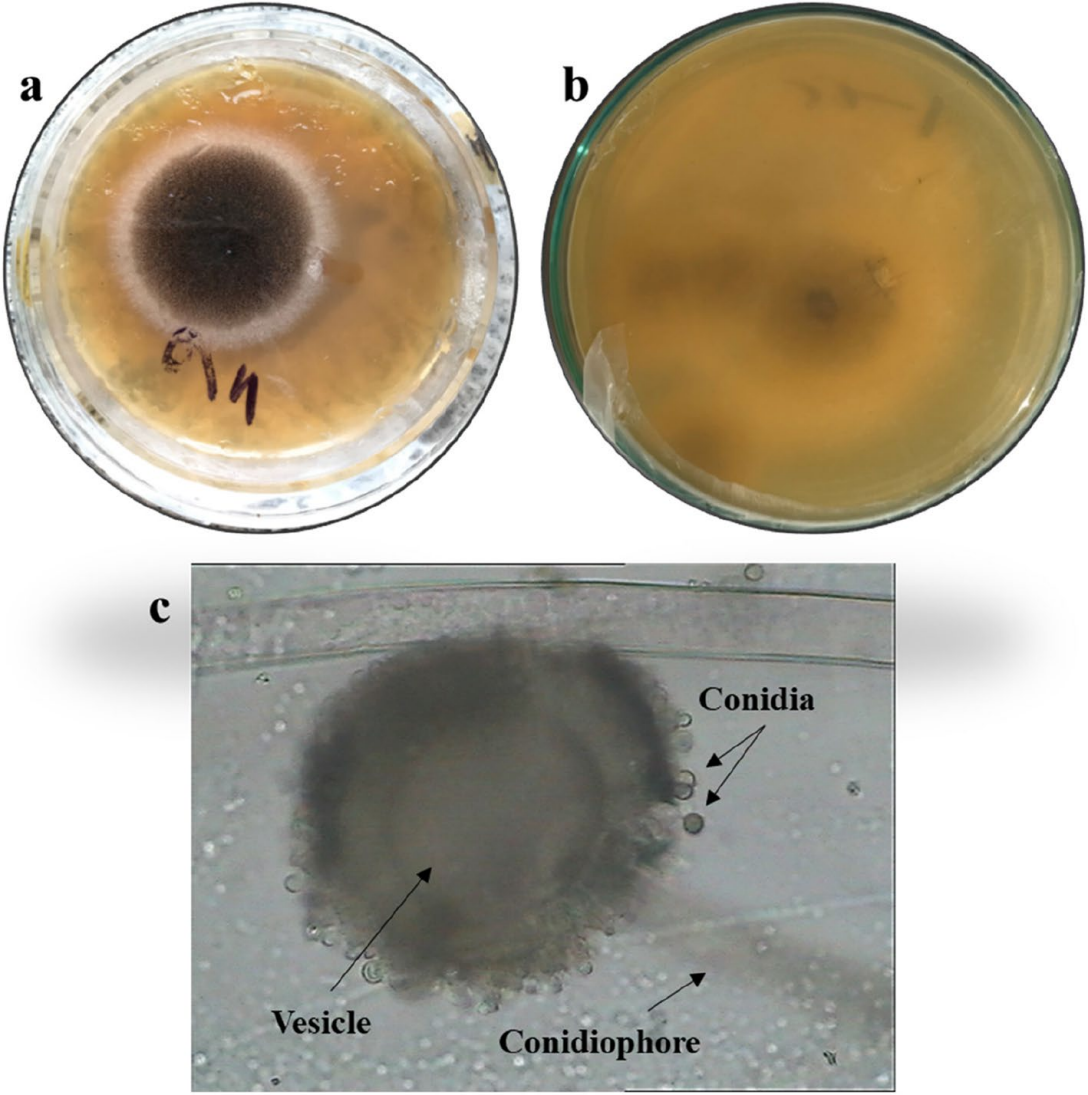
*Aspergillus japonicus* a colony on MEA, **b** colony reverse, **c** microscopic characteristics

**Fig. 5:**
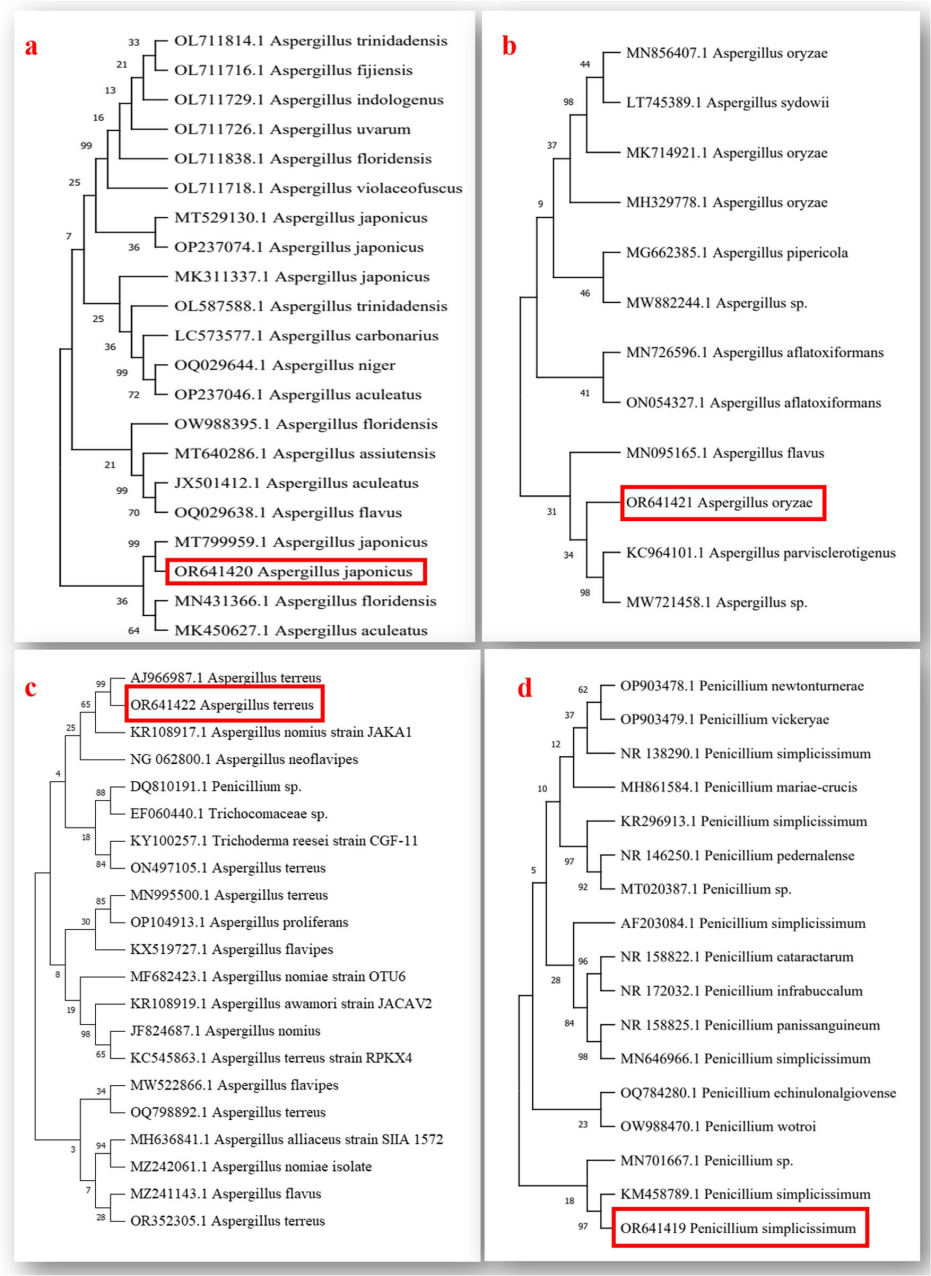
Phylogenetic tree analysis for **a** *Aspergillus japonicus,* **b** *Aspergillus oryzae*, **c** *Aspergillus terreus,* and **d** *Penicillium simplicissimum*

Species from two different genera were successfully isolated and identified in this study (Table 1 and Figs. 1, 2, 3, and 4). The findings revealed the presence of three distinct species belonging to the genus *Aspergillus* and one species from the genus *Penicillium.* To facilitate further reference, the 18S-rDNA sequences for the isolated species have been deposited in the GenBank at the National Centre for Biotechnology Information (NCBI). The accession numbers for these sequences are as follows: OR641419 for *Penicillium simplicissimum*, OR641420 for *Aspergillus japonicus*, OR641421 for *Aspergillus oryzae*, and OR641422 for *Aspergillus terreus*, respectively. The phylogenetic tree for each fungus is illustrated in Fig. 5.

**Table 1:** Macroscopic and microscopic characteristics of the isolated species

Isolate	Macroscopic characteristics		Microscopic characteristics		
	Surface colour	Reverse	Conidiophore	Conidia	Phialides
***Penicillium simplicissimum*** **(**Fig. 1**)**	Greyish green	Yellow–brown	Rough	Globose to ellipsoidal, 2.5–3.5 μm	Ampulliform
***Aspergillus terreus*** **(**Fig. 2**)**	Cinnamon brown	Brown	Smooth, hyaline	Globose, 1.5–2.5 μm	Biseriate
***Aspergillus oryzae*** **(**Fig. 3**)**	Greenish yellow	Greyish brown	Smooth, hyaline	Spherical to ovoidal, 4.5–8 × 4.5–7 μm	Uniseriate and Biseriate
***Aspergillus japonicus*** **(**Fig. 4**)**	Black	hyaline	Smooth, hyaline	Spherical to radiate, 4.5–6.0 × 4–5 μm	Uniseriate

### Optimization of reaction parameters for the biosynthesis of AgNPs

**Fig. 6:**
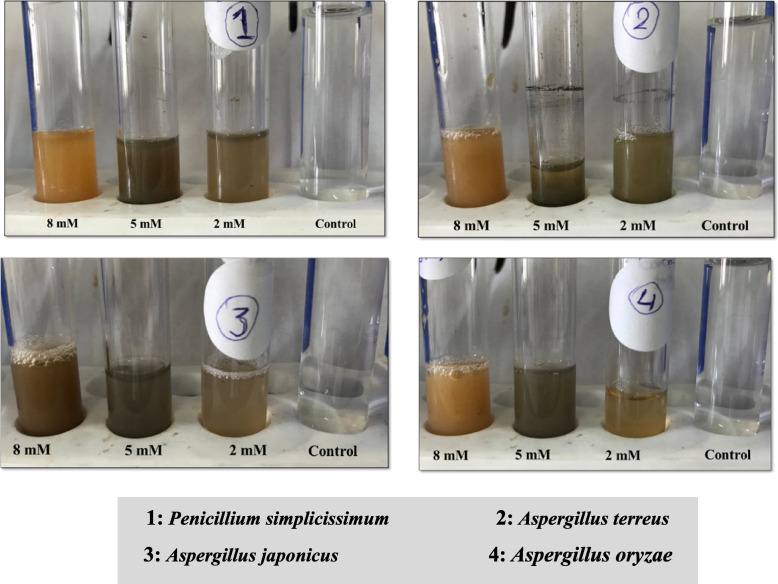
Culture filtrate of the four isolated fungi with the three most effective concentrations of silver nitrate solution

The fungal filtrates were incubated with AgNO_3_ solution in the dark in the shaker and showed a gradual increase in the color intensity of the medium throughout the incubation period, as illustrated in Fig. 6.

The change in the medium’s color was discernible through visual observation. The appearance of the brown color signified the formation of colloidal silver particles within the medium. The increase in color intensity can be attributed to the increased production of nanoparticles due to the reduction of silver.

**Fig. 7:**
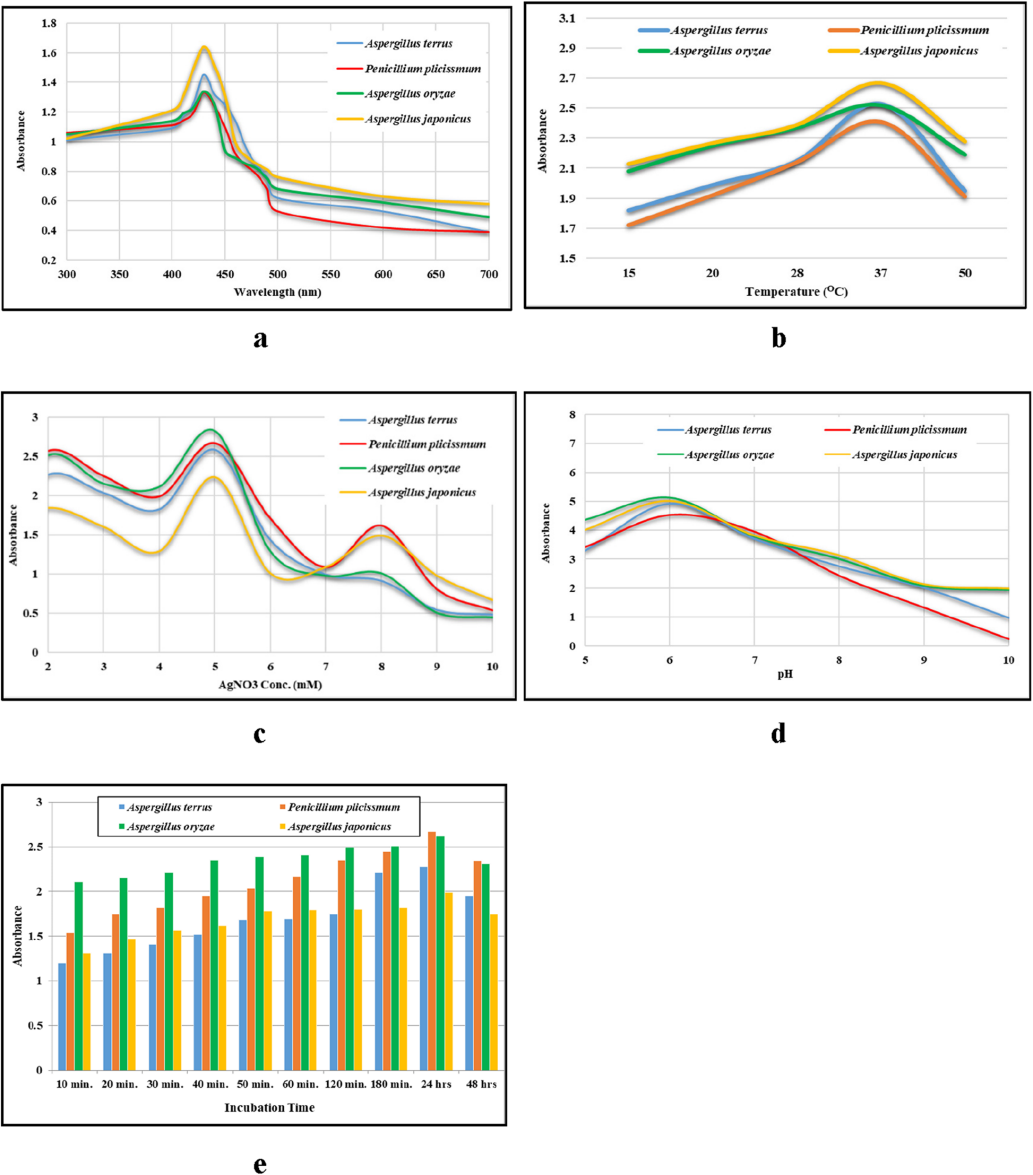
UV–Vis spectrum for optimization studies of AgNPs production at **a** different wavelength, **b** different temperatures, **c** different AgNO_3_ concentration, **d** different pH, **e** different incubation time

The obtained results indicated an elevated absorption intensity within the wavelength range of 400 to 500 nm. Consequently, optical density measurements were taken at regular intervals to precisely determine the optimal wavelength. Notably, a prominent peak at 430 nm, denoting the surface plasmon resonance (SPR), emerged as the most suitable wavelength for the ultraviolet ray-based measurement of AgNPs in all the filtrates, as summarized in Table 2. Figure 7 illustrates that a 24-h incubation period yielded the most favorable results, while a silver nitrate concentration of 5 mM, a temperature of 37 °C, and a pH of 6 were identified as the optimal conditions for the biosynthesis of AgNPs.

**Table 2:** Antibacterial activity of the Myco-biosynthesized AgNPs using four marine fungal extracts against tested pathogenic bacteria

Antibacterial activity (mm)								
	*E. faecalis*	*E. coli*	*K. pneumoniae*	MRSA	*P. vulgaris*	*S. typhi*	*S. hominis*	*S. epidermidis*
*Aspergillus terreus*								
2 mM	Null	Null	Null	Null	Null	Null	Null	Null
5 mM	Null	Null	Null	Null	18 ± 2^c^	11 ± 1^f^	12 ± 2^d^	Null
8 mM	Null	Null	Null	Null	15 ± 2^d^	14 ± 1^de^	14 ± 2^d^	Null
*Penicillium simplicissmum*								
2 mM	11 ± 0^b^	Null	Null	Null	23 ± 1^a^	17 ± 1^c^	24 ± 1^a^	Null
5 mM	12 ± 1^ab^	13 ± 0^b^	Null	Null	15 ± 3^c^	22 ± 2^b^	20 ± 1^b^	13 ± 2^ab^
8 mM	10 ± 2^b^	13 ± 2^b^	Null	Null	13 ± 3^abc^	21 ± 2^b^	22 ± 1^abc^	13 ± 2^ab^
*Aspergillus oryzae*								
2 mM	10 ± 1^b^	11 ± 0^c^	Null	Null	21 ± 1^ab^	15 ± 2^ cd^	23 ± 1^ab^	10 ± 1^d^
5 mM	12 ± 0^ab^	12 ± 1^bc^	11 ± 2^a^	Null	20 ± 1^bc^	22 ± 1^b^	21 ± 2^bc^	13 ± 0^ab^
8 mM	11 ± 2^b^	Null	10 ± 0^b^	Null	18 ± 1^c^	21 ± 2^b^	23 ± 2^ab^	14 ± 0^a^
*Aspergillus japonicus*								
2 mM	12 ± 2^ab^	11 ± 2^c^	Null	Null	15 ± 2^d^	12 ± 2^ef^	24 ± 1^a^	11 ± 1^ cd^
5 mM	14 ± 1^a^	15 ± 2^a^	Null	Null	23 ± 1^a^	23 ± 1^ab^	22 ± 1^abc^	12 ± 2^bc^
8 mM	12 ± 2^ab^	13 ± 1^b^	Null	Null	23 ± 0^a^	25 ± 0^a^	23 ± 0^ab^	13 ± 0^ab^

*MRSA* Methicillin Resistance *Staphylococcus aureus*, *Null* No activity. Values are the average of triplicate analyses ± standard deviation. Superscript letters are significantly different at *P* < 0.05 using Tukey’s HSD (honest significant difference) post hoc test

### TEM analysis

**Fig. 8:**
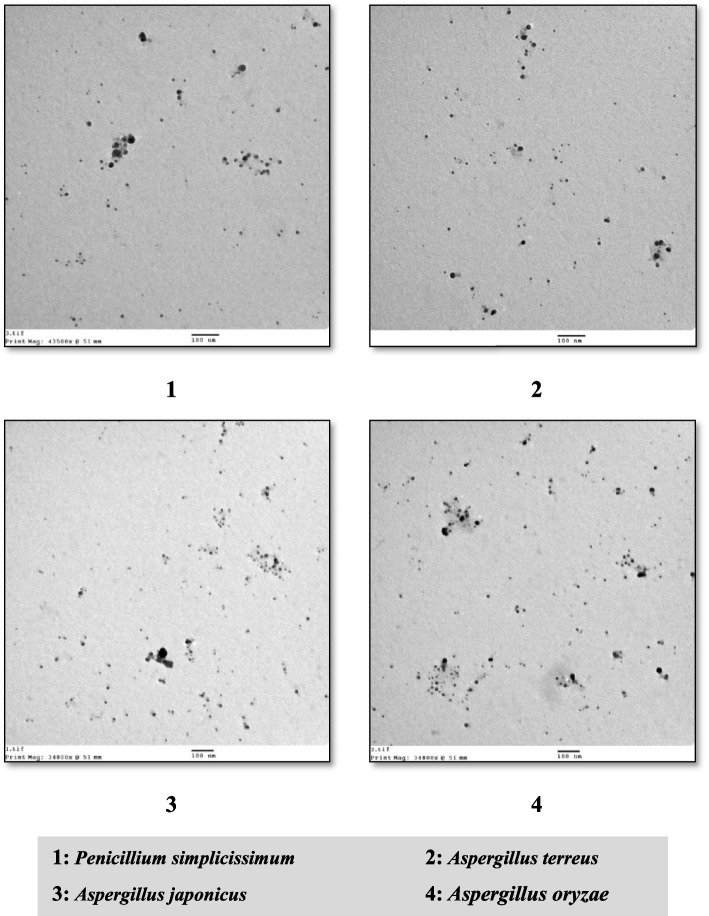
TEM micrograph of synthesized AgNPs (Scale bar: 100 nm)

High-resolution transmission electron microscopy images have provided valuable insights into the structural characteristics of the nanoparticles under investigation. These images revealed that the nanoparticles exhibited exceptional stability, demonstrating a uniform, monodisperse distribution, and a spherical morphology. The particle size ranged from 3.8 to 23 nm, as illustrated in Fig. 8.

It is worth noting that the diminutive average size of the silver nanoparticles (AgNPs) plays a pivotal role in their antimicrobial properties. Consequently, a reduction in particle size correlates with an enhancement in antimicrobial activity [13].

### EDX/SEM analysis

**Fig. 9:**
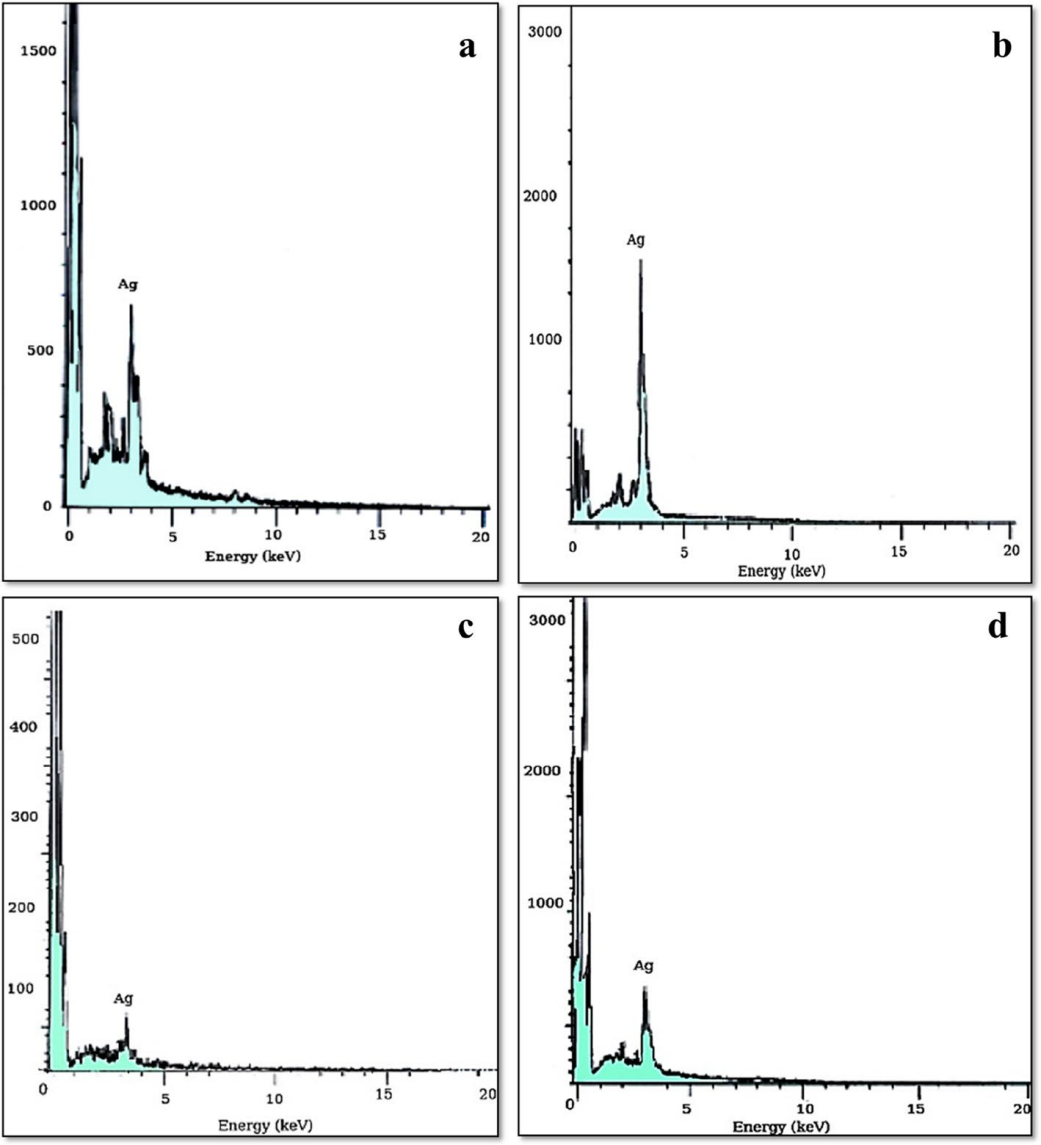
EXD Spectrum of the four biosynthesized AgNPs, **a*** Penicillium simplicissimum,* **b** *Aspergillus terreus*, **c*** Aspergillus japonicus,* **d** *Aspergillus oryzae*

The EDX analysis has revealed the emergence of a prominent peak in the silver region, specifically at 3 keV, thereby substantiating the presence of elemental silver. Consequently, this confirmed the existence of nanocrystalline elemental silver, as indicated in Fig. 9. Additionally, weak signals associated with other elements have been observed, which are likely attributed to proteins, enzymes, or the fungal biomass that are attached to the silver nanoparticles, as reported by [20].

### XRD analysis

**Fig. 10:**
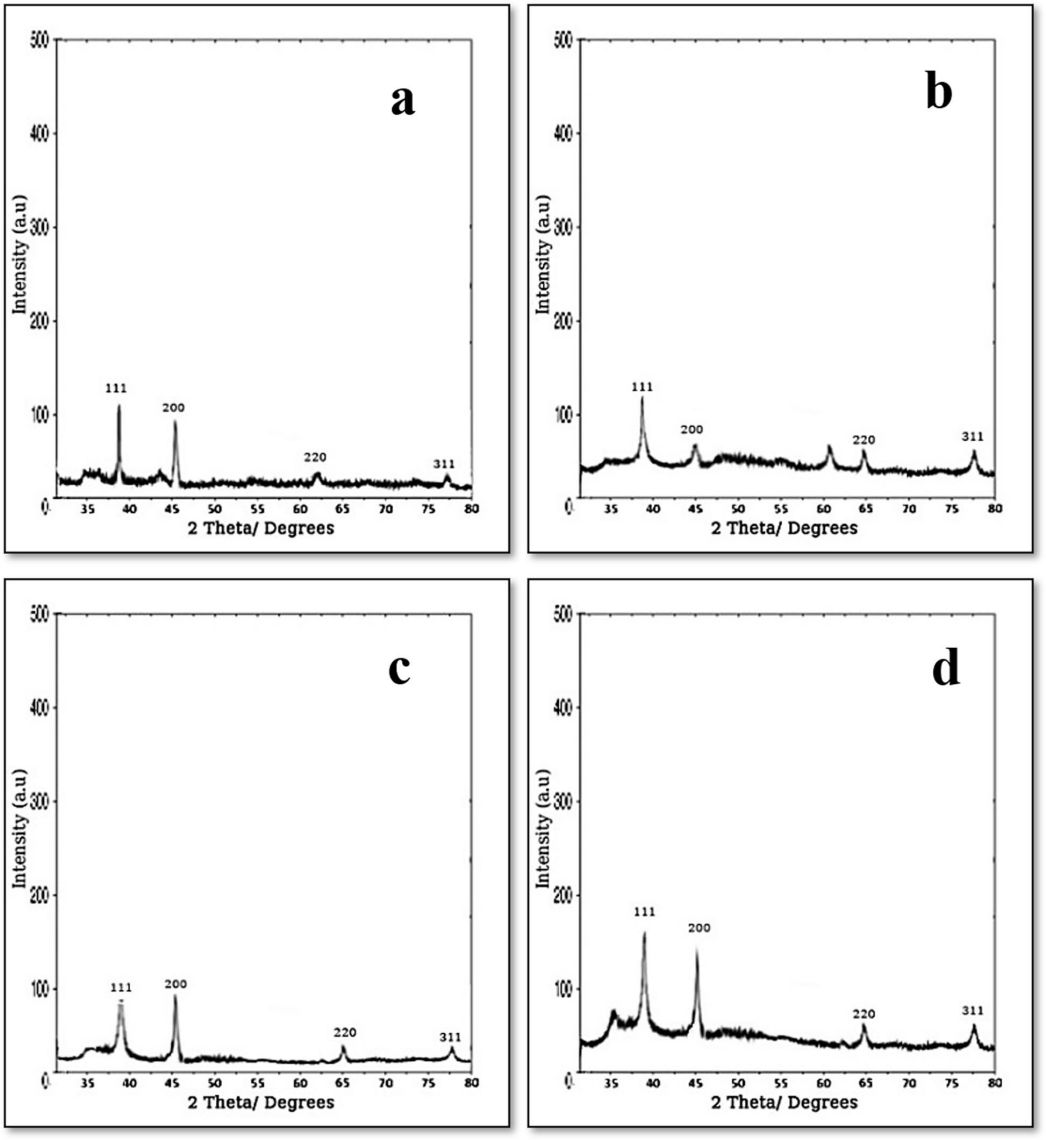
XRD patterns of the four biosynthesiezed AgNPs, **a** *Penicillium simplicissimum,* **b** *Aspergillus terreus*, **c*** Aspergillus japonicus,* **d** *Aspergillus oryzae*

The X-ray diffraction patterns, as illustrated in Fig. 10, unequivocally establish the crystalline attributes of the silver biosynthesized NPs. These patterns reveal prominent peaks spanning the angular range of 20° to 80°, which aligns with prior findings by Shaligram et al. [29]. It is noteworthy that the XRD spectra obtained from the four fungal extracts consistently exhibit distinct and robust peaks (111, 200, 220, and 311). These peaks closely coincide with Bragg’s reflections associated with silver nanocrystals, as previously reported by Dada et al. [7] and Lu et al. [17], thereby substantiating the reliability and consistency of the observed outcomes. The result was consistent with those results by Eltarahony et al. [9].

### Zeta potential

**Fig. 11:**
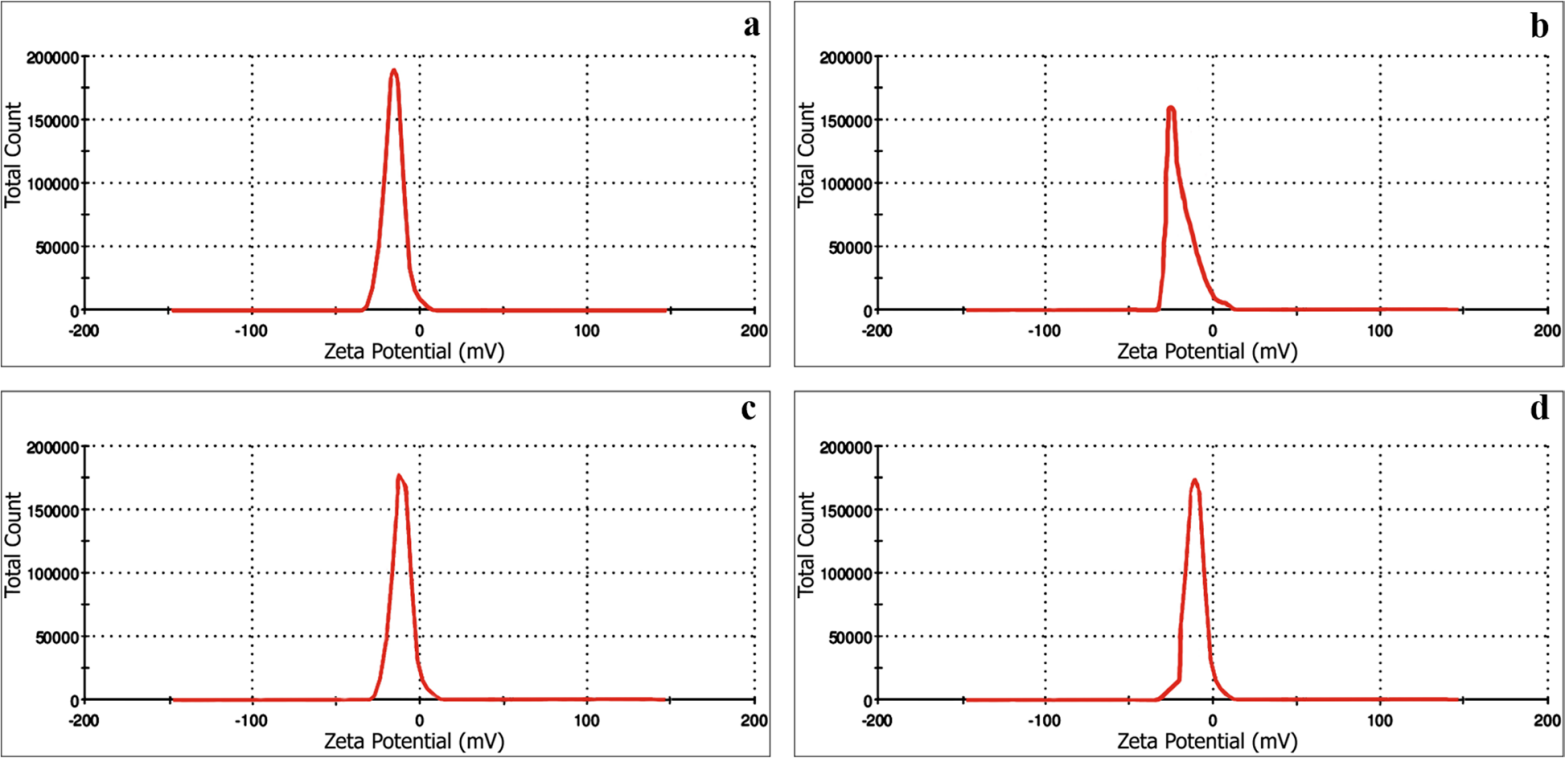
Zeta potential of the biosynthesized AgNPs, **a** *Penicillium simplicissimum,* **b** *Aspergillus japonicus*, **c** *Aspergillus oryzae,* **d*** Aspergillus terreus*

Zeta potential analysis results, as depicted in Fig. 11, revealed that the surfaces of all biosynthesized AgNPs exhibited a negative charge, with measured values of − 15, − 18, − 23, and – 25 mV for AgNPs synthesized using *Aspergillus terreus*, *Aspergillus oryzae*, *Penicillium simplicissimum*, and *Aspergillus japonicus*, respectively. These findings unequivocally affirm the stability of all biosynthesized nanoparticles. It is noteworthy that according to Eltarahony et al. [10], nanoparticles are typically considered stable when their zeta potentials fall within the range of + 30 to – 30 mV.

### FT-IR analysis

**Fig. 12:**
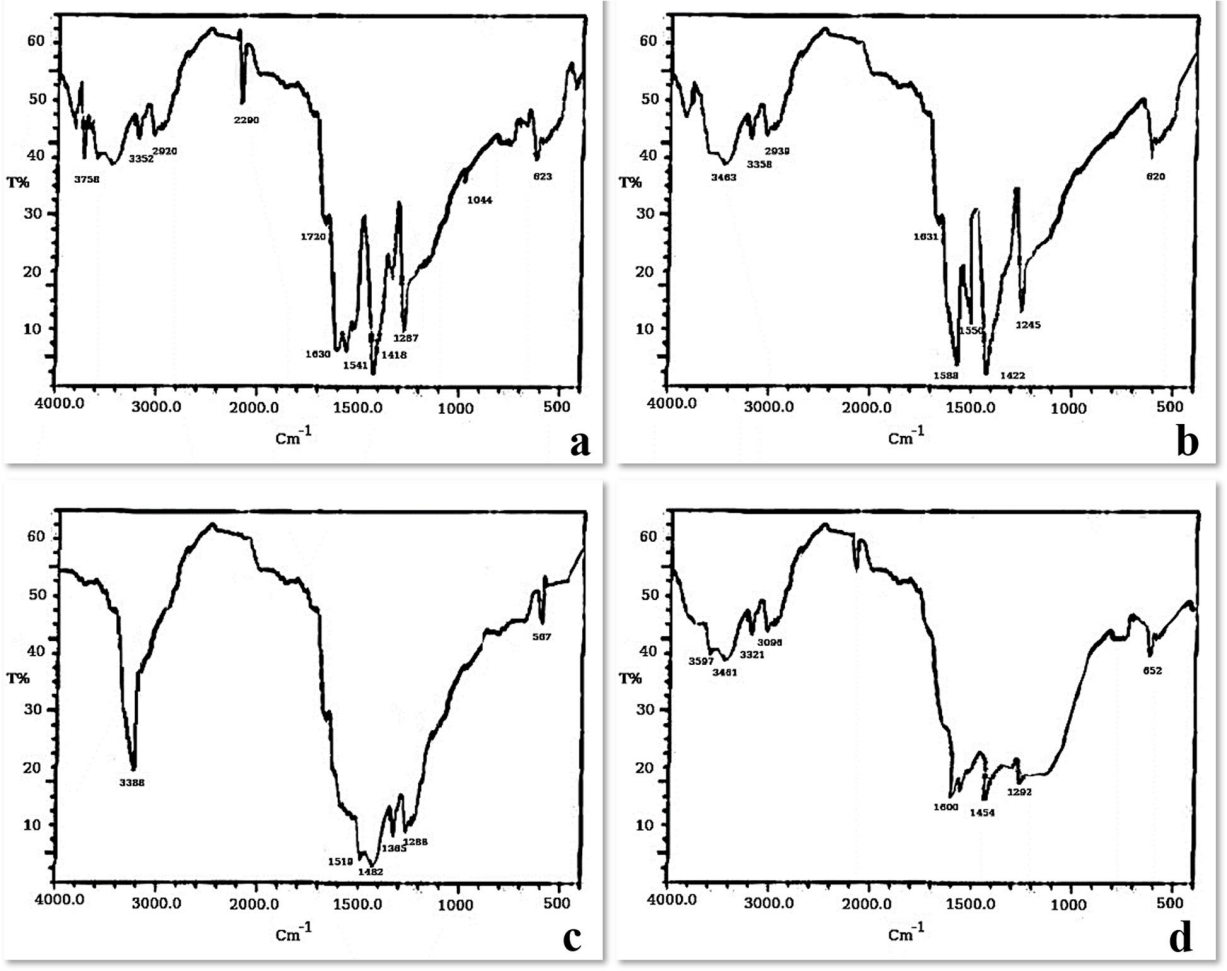
FTIR spectra of the biosynthesized AgNPs, **a*** Penicillium simplicissimum,* **b** *Aspergillus terreus*, **c*** Aspergillus japonicus,* **d** *Aspergillus oryzae*

FT-IR analyses produce an infrared absorption spectrum for identifying types of chemical bonds in a molecule which is considered as a “fingerprint”. FTIR spectra of the biosynthesized nanoparticles showed absorption peaks at 556, 762, 1349, 1451, 1562, 1620, and 3350 cm^−1^ revealing the existence of various functional groups; stretch of alkyl halides [14], phenyl group (C–H), O–H phenol [32], C–N group of amide [27], NH_2_ group [23], N–H group [33], and stretching vibration of –OH group [24] (Fig. 12).

### Antimicrobial activity results

AgNPs demonstrated notable efficacy against a wide spectrum of bacterial pathogens except MRSA, all the pathogenic yeasts, and all the pathogenic fungi except *A. niger* (Tables 2 and 3). Among the biosynthesized NPs using the four fungi, AgNPs mediated by *A. japonicus* (5 mM) had the highest antibacterial activity, while AgNPs mediated by *Penicillium simplicissmum* (8 mM) had the highest antifungal activity. Antimicrobial activity results were compatible with Eltarahony et al. [9] who highlighted the capacity of marine microorganisms to produce active metabolites along with AgNPs and cause diverse damages in several active sites such as cell wall deterioration. Antimicrobial activity results also confirmed by the results of Eltarahony et al. [10] revealed that AgNPs exhibited potential antimicrobial activity against pathogenic microorganisms which promotes their usage in different fields including wastewater purification for preventing the spread of multiple drug-resistant microbes. The study also clarified that the antimicrobial activity mechanisms include disruption of the cell wall and cell membrane, increasing the membrane permeability, causing impairment of proteins, prevention of DNA replication, and inducing oxidative stress through the production of reactive oxygen species.

**Table 3:** Antifungal activity of the Myco-biosynthesized AgNPs using four marine fungal extracts against tested pathogenic yeast and fungi

Antifungal Activity (mm)					
	*C. albicans*	*C. tropicalis*	*A. niger*	*Rhizopus oryzae*	*Penicillium expansum*
*Aspergillus terreus*					
2 mM	Null	Null	Null	10 ± 2^e^	10 ± 2^b^
5 mM	14 ± 2^a^	Null	Null	11 ± 1^cde^	11 ± 1^ab^
8 mM	12 ± 0^b^	11 ± 1^ab^	Null	13 ± 0^abc^	12 ± 0^a^
*Penicillium simplicissmum*					
2 mM	Null	Null	Null	13 ± 2^abc^	Null
5 mM	11 ± 2^a^	10 ± 1^b^	Null	14 ± 1^ab^	10 ± 0^b^
8 mM	13 ± 1^b^	11 ± 0^ab^	Null	15 ± 0^a^	12 ± 0^a^
*Aspergillus oryzae*					
2 mM	Null	Null	Null	11 ± 2^cde^	Null
5 mM	14 ± 2^a^	12 ± 1^a^	Null	12 ± 1^bcd^	Null
8 mM	10 ± 0^c^	10 ± 2^b^	Null	11 ± ^0cde^	Null
*Aspergillus japonicus*					
2 mM	Null	Null	Null	Null	Null
5 mM	Null	Null	Null	10 ± 2^de^	Null
8 mM	12 ± 0^b^	11 ± 1^ab^	Null	11 ± 2^bcd^	Null

*Null* no activity. Values are the average of triplicate analyses** ± **standard deviation. Superscript letters are significantly different at *P* < 0.05 using Tukey’s HSD (honest significant difference) post hoc test

## Conclusion

*Penicillium simplicissimum*, *Aspergillus terreus*, *Aspergillus japonicus*, and *Aspergillus oryzae* extracts, are used to synthesize AgNPs as a stabilizing and reducing agent. FTIR, EDX, TEM, zeta potential, and XRD were used for the characterization of the resulting AgNPs. FTIR spectroscopy of AgNPs confirmed the presence of functional biomolecules. TEM’s findings were supported by the SEM’s results which showed spherical morphology. AgNPs size was estimated to have a size of 3.8 to 23 nm by TEM. The elemental composition analysis through EDX displayed an intense signal of silver at 3.0 keV, affirming the silver content in the nanoparticles, while XRD analysis confirmed the crystalline nature of the AgNPs. Furthermore, the Zeta potential measurement indicated a negative charge on the biosynthesized AgNPs' surface, which is indicative of their stability. Notably, these biosynthesized AgNPs exhibited robust antimicrobial activity against a wide range of selected pathogens. In light of these findings, it is apparent that AgNPs hold significant promise and can be considered a valuable product within the field of nanobiotechnology, especially in the bioremediation of water bodies and lakes.

## Data Availability

All data and other supplementary materials are already included in the main manuscript.

## Abbreviations

AgNO_3_: Silver nitrate
AgNPs: Silver nanoparticles
EDX: Energy dispersive X-ray
FTIR: Fourier–transform infrared spectroscopy
HSD: Honest significant difference
MRSA: Methicillin Resistance *Staphylococcus aureus*
NB: Nutrient Broth
NCBI: The National Centre for Biotechnology Information
RCMB: The Regional Center of Mycology and Biotechnology
SEM: Scanning electron microscope
SPR: Surface plasmon resonance
TEM: Transmission electron microscope
XRD: X-ray diffraction
