# Correction: Mycosynthesis of silver nanoparticles using marine fungi and their antimicrobial activity against pathogenic microorganisms

**DOI:** 10.1186/s43141-023-00625-3

**Published:** 2023-12-08

**Authors:** Manar A. Basheer, Khaled Abutaleb, Nermine N. Abed, Amal A. I. Mekawey

**Affiliations:** 1 National Authority for Remote Sensing and Space Sciences (NARSS), 23 Joseph Tito Street, El‑Nozha El‑Gedida, Cairo, 1564 Egypt; 2 Agricultural Research Council, Natural Resources and Engineering (ARC-NRE), Pretoria, 0001 South Africa; 3 School of Animal, Plant and Environmental Sciences, University of Witwatersrand, Private Bag X3, Johannesburg, 2050 South Africa; 4 Faculty of Science (Girls Branch), Al-Azhar University Egypt, Cairo, 11884 Nasr City Egypt; 5 The Regional Center of Mycology and Biotechnology, Al-Azhar University, Cairo, 4434010 Egypt

**Correction: J Genet Eng Biotechnol 21, 127 (2023)**

**https://doi.org/10.1186/s43141-023-00572-z**

In the original version of this article [1], affiliations 3 and 4 were transposed due to a typesetting error. The affiliations appear correctly in this erratum, and the original article has been corrected.
